# Phosphorylation‐regulated phase separation of syndecan‐4 and syntenin promotes the biogenesis of exosomes

**DOI:** 10.1111/cpr.13645

**Published:** 2024-04-11

**Authors:** Tian Zhao, Xiaolan Yang, Guangfei Duan, Jialin Chen, Kefeng He, Yong‐Xiang Chen, Shi‐Zhong Luo

**Affiliations:** ^1^ State Key Laboratory of Chemical Resource Engineering, College of Life Science and Technology Beijing University of Chemical Technology Beijing China; ^2^ Key Laboratory of Bioorganic Phosphorus Chemistry and Chemical Biology (Ministry of Education), Department of Chemistry Tsinghua University Beijing China

## Abstract

The biogenesis of exosomes that mediate cell‐to‐cell communication by transporting numerous biomolecules to neighbouring cells is an essential cellular process. The interaction between the transmembrane protein syndecan‐4 (SDC4) and cytosolic protein syntenin plays a key role in the biogenesis of exosomes. However, how the relatively weak binding of syntenin to SDC4 efficiently enables syntenin sorting for packaging into exosomes remains unclear. Here, we demonstrate for the first time that SDC4 can undergo liquid–liquid phase separation (LLPS) to form condensates both in vitro and in the cell membrane and that, the SDC4 cytoplasmic domain (SDC4‐CD) is a key contributor to this process. The phase separation of SDC4 greatly enhances the recruitment of syntenin to the plasma membrane (PM) despite the weak SDC4‐syntenin interaction, facilitating syntenin sorting for inclusion in exosomes. Interestingly, phosphorylation at the only serine (179) in the SDC4‐CD (Ser179) disrupts SDC4 LLPS, and inhibited phosphorylation or dephosphorylation restores the SDC4 LLPS to promote its recruitment of syntenin to the PM and syntenin inclusion into exosomes. This research reveals a novel phosphorylation‐regulated phase separation property of SDC4 in the PM through which SDC4 efficiently recruits cytosolic syntenin and facilitates the biogenesis of exosomes, providing potential intervention targets for exosome‐mediated biomedical events.

## Graphical abstract

This research uncovers a novel phosphorylation‐regulated phase separation property of SDC4 on the PM by which SDC4 realizes efficient recruitment of cytosolic syntenin and thereby facilitates the biogenesis of exosomes, providing potential intervention targets for exosome‐involved biomedical events.

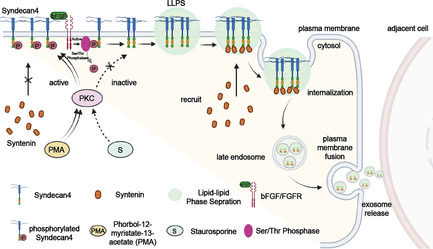

## 1 INTRODUCTION

Exosomes are secreted into the extracellular space to mediate certain forms of intercellular communication^1^, ^2^ and pathophysiological processes, such as neurodegeneration,^3^ cardiovascular disease^4^ and tumour progression.^5^ The biogenesis of exosomes starts with extracellular vesicle (EV) budding from plasma membrane (PM)‐formed endosomes to form multivesicular bodies (MVBs), which then fuse with the cell PM to secrete intraluminal vesicles (ILVs).^6^ The protein–protein interaction (PPI) network of certain membrane‐anchored receptors and signalling proteins is involved in exosomes biogenesis.^2^, ^7^, ^8^ Syndecan‐4 (SDC4), a transmembrane proteoglycan, is a central mediator of cell adhesion, migration, proliferation and endocytosis.^9^ Moreover, the C‐terminal cytoplasmic domain of SDC4 (SDC4‐CD) binds weakly to the PDZ domains of cytosolic syntenin.^10^, ^11^, ^12^ Notably, syntenin‐ and SDC4‐relevant cargos have been shown to support ILV formation. ALIX (also known as PDCD6IP) and ESCRT (also known as PDCD6IP) can interact with syntenin to participate in endosomal membrane budding and exosome formation.^12^ However, the molecular mechanism by which the relatively weak binding of syntenin to SDC4 efficiently sorts syntenin for inclusion into exosomes remains unclear.

Liquid–liquid phase separation (LLPS) is driven by multivalent weak interactions,^13^ which is a fundamental mechanism for organizing intracellular space.^14^, ^15^, ^16^, ^17^ Many membrane receptors form clusters on the cell membrane through LLPS, which is driven by higher‐order oligomerization.^18^, ^19^ These phase‐separated clusters recruit their cytoplasmic binding partners into these cluster‐formed compartments via multivalent interactions, thereby promoting downstream signal transduction in cells.^20^, ^21^, ^22^ We found that the SDC4‐CD contains a low‐complexity domain (LCD, residues 173–198), which is commonly associated with LLPS. In addition, previous reports revealed that the oligomerization of SDC4 in the PM^23^ facilitated the recruitment of certain intracellular proteins to the clusters.^24^ Thus, it is necessary to explore whether SDC4 undergoes LLPS in the cell PM to enhance its association with cytosolic syntenin, which might facilitate their sorting into endosomes to promote the biogenesis of exosomes.

Posttranslational modification is a mechanism by which cells dynamically control protein interactions, assembly, and intracellular aggregation.^25^ SDC4‐CD contains only one serine residue (Ser179), the phosphorylation of which regulates SDC4‐dependent activation of cytoplasmic protein kinase C alpha (PKCα) by reducing the affinity of PKCα for PtdIns(4,5)P2 (PIP2), which inhibiting the dimer and the oligomerization of SDC4‐CD.^26^, ^27^ Previous reports suggested that PKC‐activating phorbol ester (PMA) increased only the Ser179 phosphorylation in SDC4, which failed to induce a detectable effect on the phosphorylation of threonine or tyrosine residues in the cytoplasmic tail.^28^ Chemokines such as bFGF bound to the heparan sulfate of SDC4 and induced the dephosphorylation of SDC4,^28^ which resulted in the formation of SDC4 clusters that ultimately recruited syntenin to the budding membrane.^29^, ^30^ Hence, we hypothesized that the phosphorylation at Ser179 in SDC4‐CD might regulate the specific recruitment of syntenin and subsequent exosome biogenesis by affecting the potential LLPS of SDC4.

In this study, we demonstrated the efficient in vitro or *in cellule* phase separation of SDC4‐CD and SDC4 into droplets. The SDC4 LLPS promotes the recruitment of syntenin into droplets because the C‐terminus of SDC4 and the PDZ domain of syntenin undergo a weak interaction. However, phosphorylation of Ser179 in SDC4‐CD reduces SDC4 LLPS‐forming capacity, which interferes with the recruitment of syntenin and decreases the amount of syntenin packaged into exosomes. PKC inhibitor staurosporine and bFGF induce the dephosphorylation of SDC4 at Ser179, driving a phase transition of the SDC4‐syntenin complex that is required for efficient syntenin secretion within exosomes. We propose a novel working model to explain the phosphorylation‐regulated phase separation of SDC4 and the efficient recruitment of cytosolic syntenin to facilitate exosome biogenesis.

## 2 RESULTS

### 2.1 Phase separation of SDC4 is regulated by phosphorylation

**FIGURE 1:**
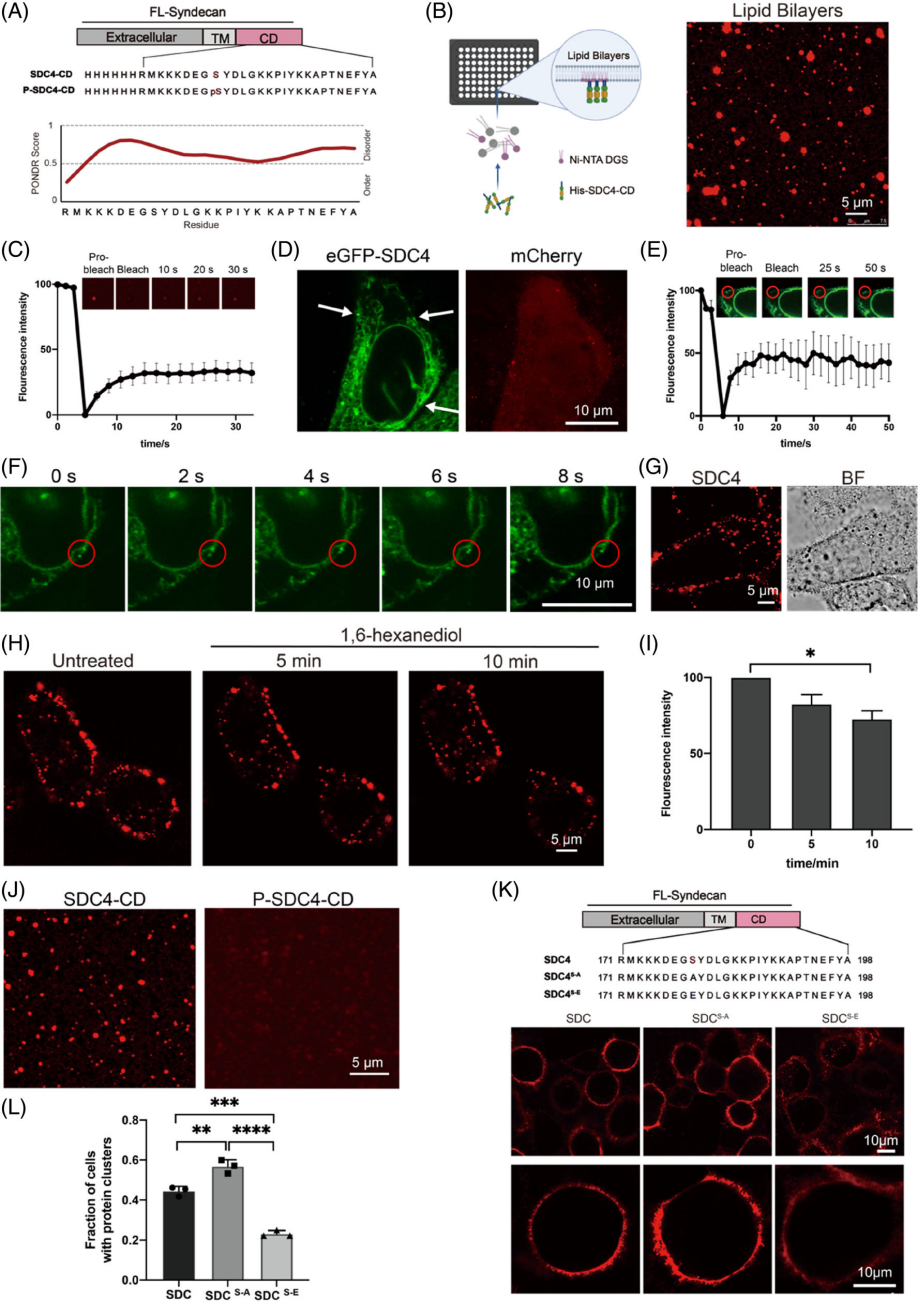
Phosphorylation of Ser179 weakened SDC4 Phase Separation. (A) Schematic diagram and amino acid sequences of SDC4‐CD and P‐SDC4‐CD; level of structural disorder (as determined using PONDR^32^). (B) Confocal fluorescence microscopy images of the assembly status of Cy3‐labelled SDC4‐CD on supported lipid bilayers (SLBs). Scale bar = 5 μm. (C) Fluorescence recovery after photobleaching (FRAP) of the droplets formed by SDC4‐CD on SLBs. (D) Confocal laser scanning microscopy (CLSM) images of eGFP‐tagged endogenous SDC4 phase separation on the PM of HeLa cells. (E) FRAP assay showing the fluorescence recovery of endogenous SDC4 condensates on the plasma membrane (PM); *n* = 3 biologically independent samples, and the data are presented as the mean values ± SEMs. (F) The time‐lapse imaging of endogenous SDC4. (G) Confocal laser scanning microscopy (CLSM) images of SDC4 phase separation on the PM of HeLa cells. An anti‐SDC4‐ecto antibody and a Cy3‐labelled secondary antibody were used to detect the location of SDC4. Scale bar = 5 μm. (H) Fluorescence images showing the disruption of the phase separation of SDC4 on the PM after 10% (w/v) 1,6‐hexanediol treatment. (I) Graph showing the fluorescence intensity of SDC4 on the PM after 10% (w/v) 1,6‐hexanediol treatment. *n* = 3 biologically independent samples, and the data are presented as the mean values ± SEMs (*p < 0.05). (J) Fluorescence images showing that Cy3‐labelled P‐SDC4‐CD formed a negligible number of droplets on the SLBs compared with the number formed by Cy3‐labelled SDC4‐CD. Scale bar = 5 μm. (K) The CLSM images showing droplets formed by SDC4, SDC4^S‐A^ and SDC4^S‐E^ on the PM; Schematic diagram and amino acid sequences of SDC4, SDC4^S‐A^ and SDC4^S‐E^. (L) Quantification of the fraction of SDC4, SDC4^S‐A^ and SDC4^S‐E^ overexpressed cells with protein clusters on the PM; *n* = 3 biologically independent samples, and the data are presented as the mean values ± SEMs (**p < 0.01, ***p < 0.001, ***p < 0.0001).

Phase separation is driven by oligomerization/polymerization of proteins through their modular binding domains.^21^ Previous biochemical studies indicated that oligomerization of SDC4‐CD is important for SDC4‐mediated signal transduction.^24^, ^31^ Notably, SDC4‐CD residues have been highly conserved throughout evolution (Supplementary Materials Figure S1). A PONDR analyses^32^ predicted a LCD (residues 173–198) within the SDC4‐CD sequence (Figure 1A), suggesting that this intrinsically disordered region might enable protein assembly and concomitant phase separation. In order to realize the analysis of SDC4's phase separation on the PM in vitro, we constructed the supported lipid bilayers (SLBs). Then, we attached Cy3‐labelled His_6_‐SDC4‐CD to the SLBs, which contained 5% Ni‐1,2‐dioleoyl‐sn‐glycero‐3‐[(N‐(5‐amino‐1‐carboxypentyl) iminodiacetic acid) succinyl] (Ni‐NTA DGS) and 95% 1‐palmitoyl‐2‐oleoyl‐glycero‐3‐phosphocholine (POPC). Membrane‐bound SDC4‐CD formed droplets on the SLBs, and the droplets were homogeneous and fluid, as demonstrated by the fluorescence images from confocal laser scanning microscopy (CLSM) and the results of fluorescence recovery after photobleaching (FRAP) assay (Figure 1B,C). Besides, we incubated different concentrations of Cy3‐labelled SDC4‐CD with 10% PEG‐8000, the CLSM images showed that Cy3‐labelled SDC4‐CD formed liquid‐like droplets in PBS buffer and became denser as the SDC4‐CD concentration was increased (Supplementary Materials Figure S1). The liquid behaviour analysis was supported by the FRAP results (Supplementary Materials Figure S1). In addition, NaCl and 1,6‐hexanediol are two widely used methods to analyse the nature of the molecular interactions implicated in protein phase separation^33^, ^34^, ^35^, ^36^ Treatment with NaCl or 1,6‐hexanediol caused SDC4‐CD puncta to disassemble with the increase of the concentration, which further illustrates that SDC4‐CD has the potential to phase separate (Supplementary Materials Figure S1). Moreover, to analyse the phase separation of endogenous SDC4 in living cells, we tagged endogenous SDC4 with N‐terminal eGFP by using CRISPR/Cas9 knock‐in technique (eGFP‐ SDC4 KI) in HeLa cells (Supplementary Materials Figure S2a,b). We found that SDC4 in HeLa cells could form droplets (Figure 1D). The results of FRAP assay showed the fluorescence recovery of endogenous SDC4 condensate (Figure 1E) and the time‐lapse images demonstrated a progressive fusion of endogenous SDC4 condensates as time elapsed, exhibiting characteristics similar to liquid droplets (Figure 1F). Meanwhile, the CLSM images showed that the Cy3‐labelled secondary antibody bound full‐length SDC4 overexpressed on the PM of HeLa cells and formed droplets (Figure 1G) that were disassembled in a time‐dependent manner after the treatment of 10% (w/v) 1,6‐hexanediol (Figure 1H). We have also used quantitative method^37^ to analysed membrane‐based images and quantified the fluorescence intensity of droplets decrease on the PM (Figure 1I). These findings suggested that SDC4 may undergo LLPS at the cell membrane.

Ser179 is the only serine in SDC4‐CD, its phosphorylation has been previously shown to destabilize SDC4‐C oligomers^24^ and attenuate signal transduction.^38^ To investigate the regulatory effect of Ser179 phosphorylation on SDC4‐CD LLPS, we synthesized unphosphorylated SDC4‐CD and phosphorylated SDC4‐CD (P‐SDC4‐CD) (Figure 1A). Next, CLSM fluorescence images were captured and showed that Ser179 phosphorylation disrupted the formation of SDC4‐CD droplets on SLBs and in vitro (Figure 1J) (Supplementary Materials Figure S2c). In addition, we constructed two full‐length SDC4 mutants (SDC4^S‐E^ and SDC4^S‐A^) and transfected them into HeLa cells. Then, we used an anti‐SDC4‐ecto antibody and a Cy3‐labelled second antibody to determine the location of SDC4, in which Ser179 was respectively replaced with glutamic acid, a general hydrolysis‐resistant mimic of phosphorylated serine, or a non‐phosphorylable alanine. The CLSM images revealed that cells overexpress SDC4^S‐A^ formed much cluster to the PM than SDC4 (Figure 1K,L), while cells overexpress SDC4^S‐E^ formed fewer clusters than SDC4. These results demonstrate that phosphorylation of Ser179 suppresses the phase separation of SDC4 on PM.

### 2.2 Phase separation of SDC4 recruits syntenin to the PM

**FIGURE 2:**
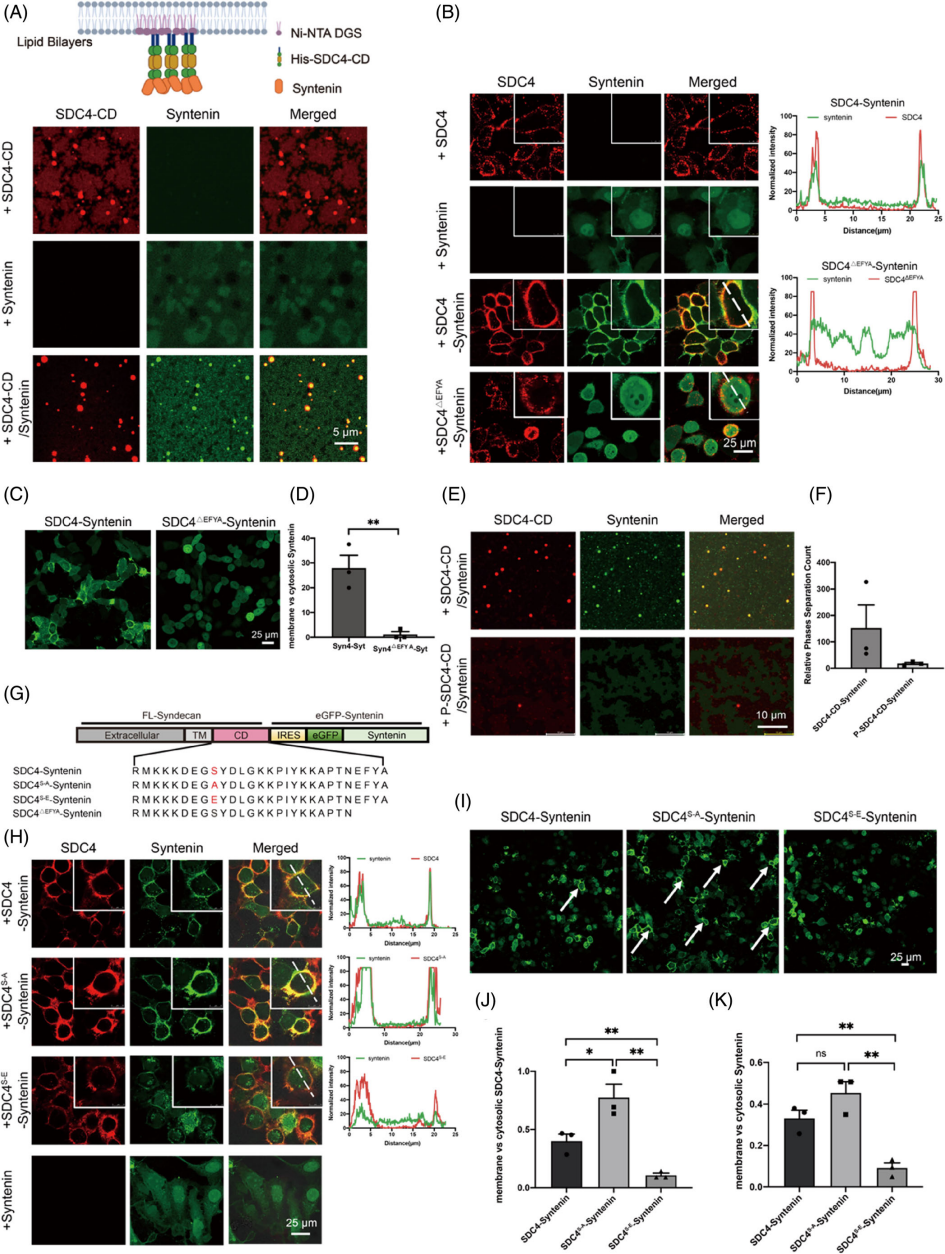
Phosphorylation of SDC4‐CD decreased syntenin recruitment to the PM. (A) Confocal microscopy images of the assembly of Cy3‐labelled SDC4‐CD with iFluor 488‐labelled syntenin on supported lipid bilayers (SLBs). Scale bar = 5 μm. (B) Confocal microscopy images of SDC4, SDC4^S‐A^, SDC4^S‐E^ and SDC4^△EFYA^ with eGFP‐tagged syntenin during cophase separation on the PM; eGFP‐tagged syntenin was expressed as the control. An anti‐SDC4‐ecto antibody and a Cy3‐labelled secondary antibody was used to detect the location of SDC4. Fluorescence colocalization was analysed by ImageJ software. (C) Without an antibody recognizing SDC4, confocal microscopy images showing eGFP‐tagged syntenin recruitment to the PM of SDC4 and syntenin, SDC4^△EFYA^ and syntenin‐coexpressing HeLa cells. Scale bar = 25 μm. (D) The number of cells recruited syntenin to the cell membrane were counted. (E) Fluorescence images showing that 100 μM Cy3‐labelled P‐SDC4‐CD with 25 μM iFluor 488‐labelled syntenin triggered profoundly fewer droplets than were formed with treatment by the same molar concentration of Cy3‐labelled Syn‐CD and iFluor 488‐labelled syntenin on SLBs. Scale bar = 10 μm. (F) Histogram showing the number of SDC4‐CD‐syntenin droplets. (G) Schematic diagram and amino acid sequences of the SDC4 and syntenin coexpression plasmid containing unphosphorylated SDC4 and a mimic of phosphorylated SDC4 with eGFP‐tagged syntenin (SDC4^S‐A^‐syntenin and SDC4^S‐E^‐syntenin). Functional domains are shown in boxes. (H) Immunofluorescence (IF) assay showing that SDC4^S‐E^ exhibited a weaker interaction with syntenin and that SDC4^S‐A^ recruited a significant amount of syntenin to the plasma membrane (PM). Fluorescence colocalization was analysed with ImageJ software. An anti‐SDC4‐ecto antibody and a Cy3‐labelled secondary antibody were used to detect the location of SDC4. (I) Without an antibody recognizing SDC4, confocal microscopy images of eGFP‐tagged syntenin recruitment to the PM in SDC4 and syntenin, SDC4^S‐A^ and syntenin, SDC4^S‐E^ and syntenin‐coexpressing HeLa cells. Scale bar = 25 μm. (J) The number of cells that recruited SDC4 and syntenin to the cell membrane, as shown in Figure 2H, were counted. (*p < 0.05, **p < 0.01). (K) The number of cells that recruited eGFP‐tagged syntenin to the cell membrane is shown in Figure 2I (**p < 0.01).

Previous biochemical studies revealed that via the syntenin PDZ domain, syntenin recognized the C‐terminal PDZ‐binding motif (PBM) of SDC4, with a low binding affinity that was similar that of stargazin with PSD‐95 (in the tens of micromolar range).^34^ We applied both Cy3‐labelled SDC4‐CD and iFluor 488‐labelled syntenin to SLBs and observed that syntenin formed micrometre‐sized clusters facilitated by SDC4‐CD, as shown in CLSM images (Figure 2A). Moreover, the FRAP results showed that the syntenin condensate was rapidly re‐formed (Supplementary Materials Figure S2d), indicating by the co‐phase separation of syntenin with SDC4. We also found that the SDC4 LLPS leads to the recruitment of syntenin and its subsequent enrichment into condenses in PBS buffer (Supplementary Materials Figure S3a), and employed the FRAP assay, varied concentrations of NaCl, and a 10% solution of 1,6 hexanediol to further investigated the fluidity of the SDC4‐syntenin condensates (Supplementary Materials Figure S3b–e). Moreover, a molecular dynamics simulation predicted that the association between SDC4 and syntenin primarily occurs via hydrophobic and charge–charge interactions (Supplementary Materials Figure S3f). To exclude the influence of the His‐tag, liquid droplet formation was confirmed with rhodamine B‐labelled syndecan‐4 (SDC4)‐CD (Supplementary Materials Figure S4). These results showed that both hydrophobic and charge–charge interactions mediated the phase separation between SDC4 and syntenin. Next, we sought to determine whether SDC4 recruits syntenin from the cytosol to form droplets on the cell membrane. Hence, we overexpressed full‐length SDC4 and eGFP‐tagged syntenin in HeLa cells and analysed the location of syntenin with a Cy3‐labelled secondary antibody. CLSM images showed that a significant amount of syntenin was recruited to the PM by SDC4, but syntenin largely remained in the cytosol when only syntenin was overexpressed in cells (Figure 2B). In addition, the FRAP analysis showed that the SDC4 and syntenin levels were dynamic in the condensate clusters (Supplementary Materials Figure S5a). Treatment with 10% (w/v) 1,6‐hexanediol caused SDC4‐syntenin puncta to disassemble in a time‐dependent manner (Supplementary Materials Figure S5b,c).

To further investigate the effect of the SDC4 PDZ‐binding motif (EFYA) on the recruitment of syntenin to the PM, we constructed an EFYA‐deleted SDC4 mutant (SDC4^△EFYA^) (Figure 2G). CLSM images demonstrated that SDC4^△EFYA^ did not recruit syntenin to the PM (Figure 2B). To exclude the influence of the anti‐SDC4‐ecto antibody, we analysed only eGFP‐syntenin recruitment to the PM in HeLa cells overexpressing both SDC4 and syntenin in the absence of the anti‐SDC4‐ecto antibody. The results clearly demonstrated that the SDC4 EFYA and syntenin PDZ interaction played a decisive role in eGFP‐syntenin recruitment to the PM (Figure 2C,D).

### 2.3 Phosphorylation of SDC4 interferes with the recruitment of syntenin to the PM

Next, to examine the effect of phosphorylation in SDC4‐CD on syntenin recruitment to the PM, we incubated iFluor 488‐labelled syntenin with Cy3‐labelled SDC4‐CD or the phosphorylated counterpart (P‐SDC4‐CD) with SLBs. As shown in the CLSM images (Figure 2E,F), phosphorylation of SDC4‐CD at Ser179 not only disrupted the formation of LLPS droplets but also significantly suppressed the recruitment of syntenin to SDC4. Ser179 localized within the disordered region of SDC4‐CD, which is upstream and far from the PDZ‐binding motif recognized by syntenin, suggesting that phosphorylation at Ser179 suppressed the recruitment of syntenin to SDC4, probably by disrupting the formation of SDC4 condensates. In addition to these in vitro assays, we generated HeLa cells coexpressing SDC4^S‐A^ and eGFP‐syntenin or SDC4^S‐E^ and eGFP‐syntenin with an internal ribosome entry site (IRES) (Figure 2G). The nonphosphorylatable SDC4 mutant recruited syntenin to the droplets on the PM much more efficiently than the phosphorylated SDC4 mutant (Figure 2H,J). Without incubation with the anti‐SDC4‐ecto antibody, we found that eGFP‐tagged syntenin was recruited to the PM (Figure 2I,K), and a FRAP assay revealed that the fluidity of the droplets with and without exposure to the anti‐SDC4‐ecto antibody was the same (Supplementary Materials Figure S5d).

**FIGURE 3:**
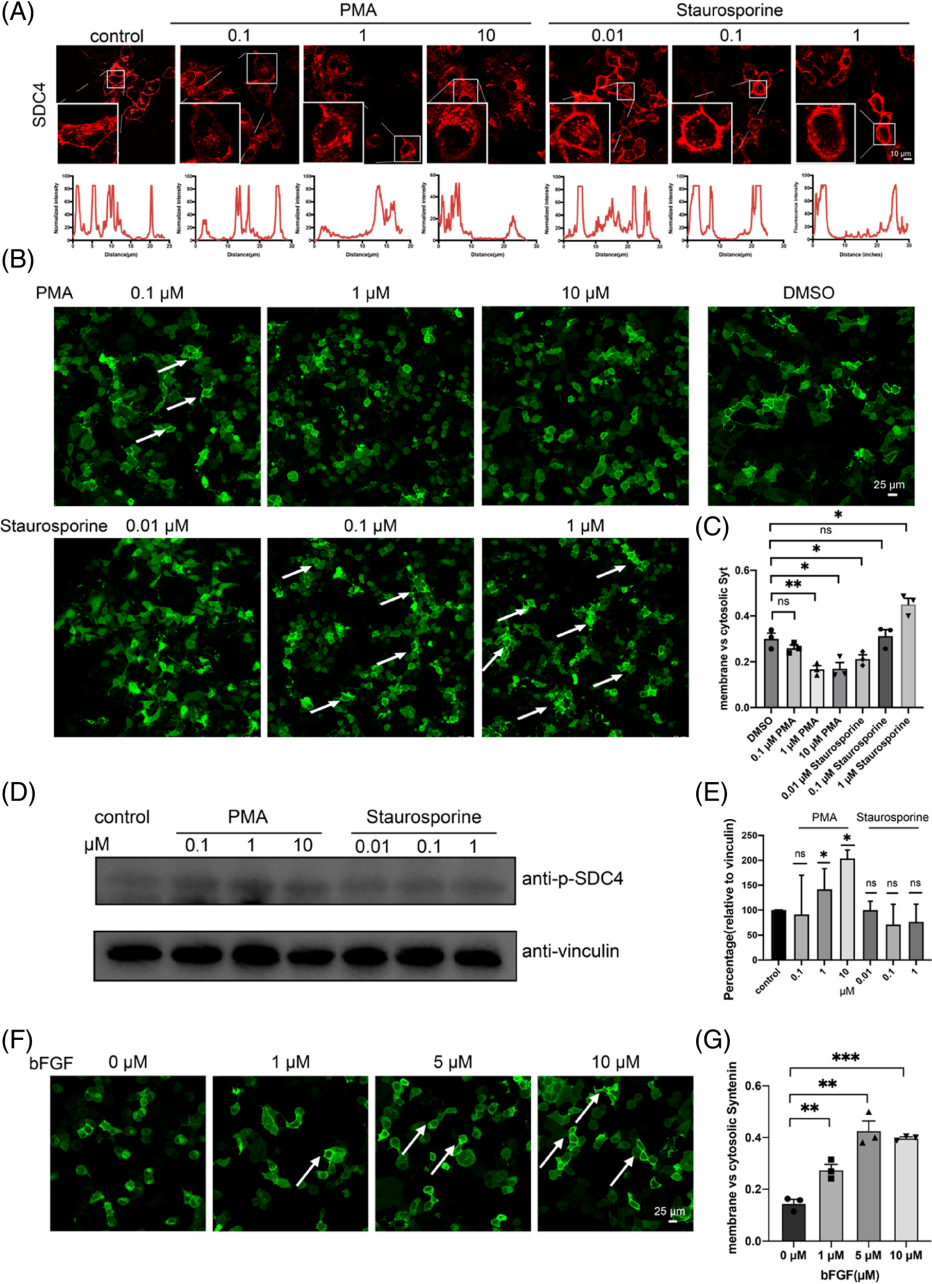
Dephosphorylation of SDC4 increased SDC4 phase sepration and the recruitment of syntenin on the plasma membrane (PM). (A) Confocal laser scanning microscopy (CLSM) images of Staurosporine and PMA regulate SDC4 clusters on the PM of HeLa cells. An anti‐SDC4‐ecto antibody and a Cy3‐labelled secondary antibody were used to detect the location of SDC4. (B) SDC4‐eGFP‐syntenin cells were stimulated with PKC‐activating phorbol ester (PMA) and staurosporine. Dimethyl sulfoxide (DMSO) was used as the control. (C) For the quantification of the subcellular localization of syntenin stimulated with PMA or staurosporine, the number of cells that have syntenin in the cytoplasm and at the plasma membrane was analysed. The y‐axis represents the ratio of the number of cells that have syntenin on the membrane to those in the cytoplasm. The results were calculated in 3 independent experiments (**p < 0.01). (D) Western blot showed that PMA increased the phosphorylation level of SDC4, but staurosporine did not yield an obvious outcome. (E) Quantification of the level of SDC4 phosphorylation (*p < 0.05). (F) SDC4‐eGFP‐syntenin cells were stimulated with bFGF. (G) For the quantification of the subcellular localization of syntenin stimulated with bFGF, the number of cells that have syntenin in the cytoplasm and at the plasma membrane was analysed. The y‐axis represents the ratio of the number of cells that have syntenin on the membrane to those in the cytoplasm. The results were calculated in 3 independent experiments (**p < 0.01, ***p < 0.001).

Previous reports revealed that PKC catalysed the phosphorylation of Ser179 in SDC4‐CD.^23^, ^28^, ^31^, ^39^ Therefore, we treated SDC4 and syntenin coexpressing HeLa cells with PMA or with staurosporine (a PKC inhibitor) separately. The CLSM results indicated that PMA interfered with the phase separation of SDC4 on the cell membrane (Figure 3A) and reduced the recruitment of syntenin to the cell membrane. However, the effect was opposite with the addition of the PKC inhibitor staurosporine (Figure 3B,C). We also detected the regulation of PMA or staurosporine on SDC4 phosphorylation in cells by western blot. The results showed that PMA could enhance SDC4 phosphorylation in cells **(**Figure 3D,E; Supplementary Materials Figure S6). However, due to the relatively low level of SDC4 phosphorylation in unstimulated cells, the treatment of cells by staurosporine did not yield a significant change in the western blot results (Figure 3D,E; Supplementary Materials Figure S6). In addition, bFGF‐dependent serine/threonine phosphatases reduce the Ser phosphorylation of SDC4.^28^ We also conducted a western blot to test the phosphorylation of SDC4 (Supplementary Materials Figure S7a,b). Therefore, we incubated SDC4 and syntenin coexpressing HeLa cells with an increasing concentration of bFGF and then detected the cells by CLSM. The results showed that the level of syntenin gradually increased on the cell membrane with increasing bFGF concentrations, from 0 to 10 μM (Figure 3F,G). Consistent with our results obtained using a phosphorylation mimetic (Figure 2H), the PKC activator PMA increased the phosphorylation rate of Ser179 in SDC4‐CD to reduce syntenin recruitment to the cell membrane, staurosporine and the SDC4 extracellular ligand bFGF triggered the level of dephosphorylated Ser179 in SDC4‐CD, increasing syntenin recruitment to the cell membrane.

### 2.4 Dephosphorylation of SDC4 increases the level of syntenin that is packaged in secreted exosomes

**FIGURE 4:**
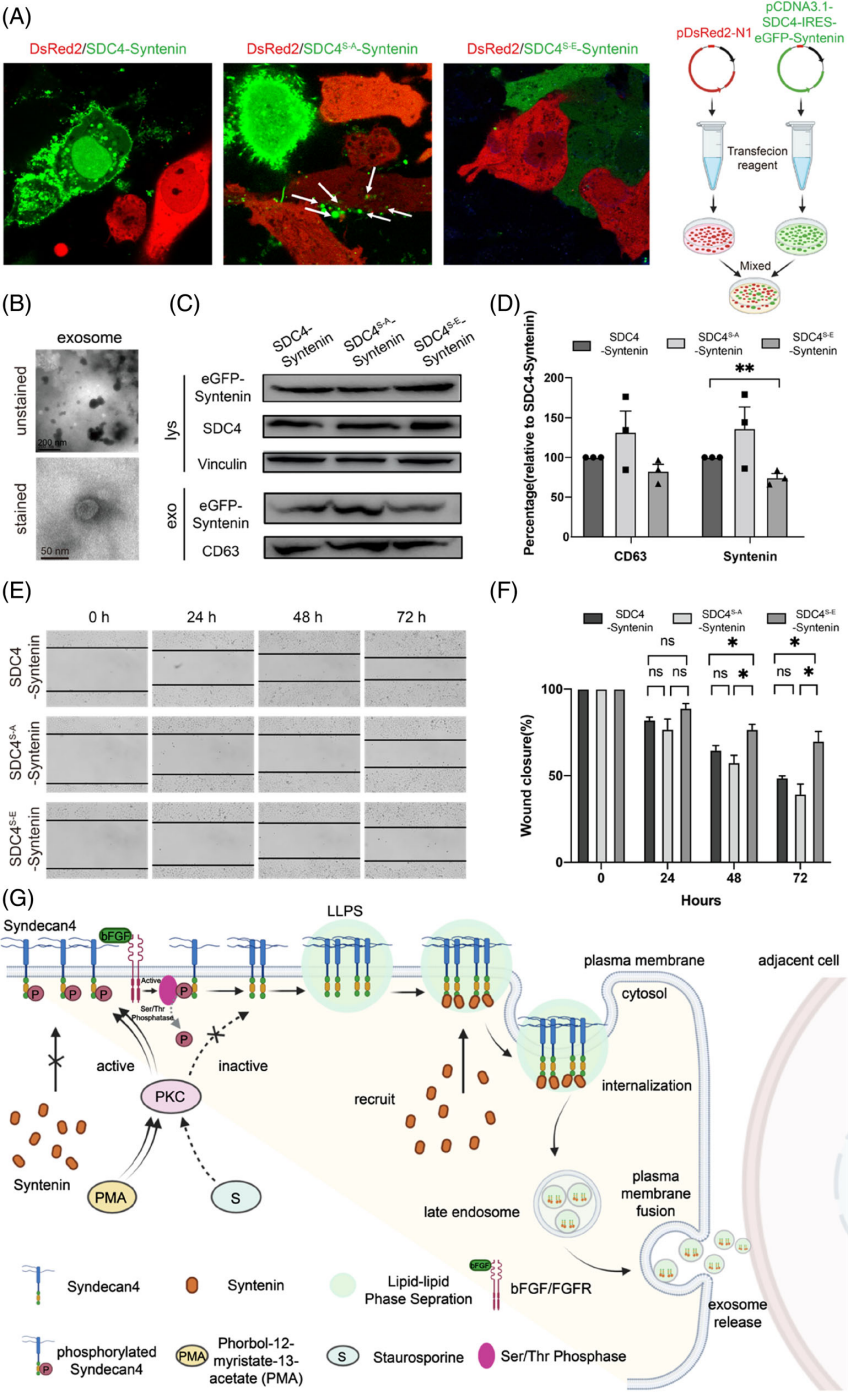
The dephosphorylation‐mimicking mutant SDC4^S‐A^‐syntenin increased the amount of syntenin in secreted exosomes and improved cell viability. (A) HeLa cells transfected with SDC4^S‐A^‐syntenin or SDC4^S‐E^‐syntenin and mixed with HeLa cells transduced with pDsRed2‐N1 for 72 h. Confocal microscopy showed that eGFP‐syntenin was detected in DsRed2‐labelled HeLa cells. (B) Transmission electron microscope (TEM) images showing the morphologies of SDC4‐syntenin exosomes. (C) Western blot showing that SDC4 phosphorylation decreased the amount of syntenin in secreted exosomes. (D) syntenin secretion from cells and in *exosomes* after SDC4 phosphorylation or dephosphorylation; *n* = 3 biologically independent samples, and the data are presented as the mean values ± SEMs. Comparisons between groups were performed via two‐tailed unpaired *t* test (**p < 0.01). (E) Percentage of the wound closed after SDC4‐syntenin, SDC4^S‐A^‐syntenin or SDC4^S‐E^‐Sy overexpression. Micrographs were taken immediately after wounding and 24, 48, and 72 h after the introduction of a wound. Black dashed lines denote wound edges. (F) Values significantly different from controls are indicated with an asterisk (*p < 0.05). The graphs show the means (with SEMs). (G) Schematic illustration showing the proposed model in which SDC4 phase separation and syntenin on the plasma membrane regulate the exosomes biogenesis and intercellular signalling activity.

Exosomes are pivotal for cell‐to‐cell communication,^40^ and their biogenesis is a complicated bioprocess involving many steps, with SDC4‐syntenin binding playing a significant role by promoting the endosomal membrane budding.^30^ Our abovementioned findings demonstrated that phosphorylation of SDC4 at Ser179 suppressed SDC4 recruitment of syntenin from the cytosol to the PM mainly by disrupting SDC4 LLPS. Therefore, to further investigate the effects of Ser179 phosphorylation on the biogenesis of exosomes, we transfected HeLa cells to overexpress SDC4 and eGFP‐syntenin, SDC4^S‐A^ and eGFP‐syntenin or SDC4^S‐E^ and eGFP‐syntenin constructs, and we transfected a pDsRed2‐N1 plasmid expressing the fluorescent protein label DsRed2 to label HeLa cells not overexpressing SDC4 or syntenin. We observed that when SDC4^S‐A^ was overexpressed, secreted exosomes transported eGFP‐syntenin to adjacent cells and that this trafficking by exosomes was disrupted when SDC4 was phosphorylated (i.e., SDC4^S‐E^ was overexpressed) (Figure 4A). During extracted from the culture medium, exosomal SDC4 co‐fractionated with syntenin and the exosomal marker CD63 (Figure 4B–D), and with vesicles, mostly with a shape and size characteristic of exosomes (Figure 4B). Moreover, a Western blot analysis showed that the level of exosomal syntenin increased with an increase in the exosomal marker CD63 when SDC4^S‐A^ and syntenin were overexpressed in HeLa cells. In contrast, this increase was not observed in cells overexpressing SDC4^S‐E^, confirming that phosphorylation of SDC4 at Ser179 plays a key regulatory role in the biogenesis of exosomes (Figure 4C,D). Exosomes carrying proteins and nucleic acids are exchanged between tumour cells and normal cells, enabling the transfer of malignant phenotypes within the microenvironment and promoting tumour cell viability and proliferation.^41^, ^42^ We observed a reduction in the motility of SDC4^S‐E^ and syntenin‐coexpressing HeLa cells, and this functional outcome was reversed by SDC4^S‐A^ and syntenin‐coexpression in HeLa cells (Figure 4E). We determined the migration distance of these three types of overexpressed cells in three independently repeated wound scratch assays, and the results were consistent with the previous results showing that cells overexpressing nonphosphorylatable SDC4 and eGFP‐tagged syntenin (SDC4^S‐A^ and syntenin‐coexpressing cells) increased cell viability (Figure 4F). To better investigate the increased cell viability of non‐phosphorylated SDC4, we repeated the wound scratch assay in CHO cells that did not express endogenous SDC4 (Supplementary Materials Figure S8a). The results better showed that nonphosphorylatable SDC4 and eGFP‐tagged syntenin coexpressed accelerated cell viability within 24 h (Supplementary Materials Figure S8b). We concluded that the phosphorylation of SDC4‐CD at Ser179 disrupted SDC4 LLPS on the cell PM, thereby preventing the recruitment of cytosolic syntenin to the PM. Upon dephosphorylation of SDC4 at Ser179, syntenin was recruited to the PM, forming cophase separation droplets with SDC4 and packaged into generated exosomes that were secreted and entered adjacent cells (Figure 4G).

## 3 DISCUSSION

On summary, we identified that the cytoplasmic domain of SDC4 mediates its phase separation to form condensates on the PM, which facilitates its recruitment of syntenin from the cytosol and that this recruitment is mediated through the interaction between the C‐terminal motif of SDC4 and the PDZ domain of syntenin. In addition, we discovered that the phosphorylation of the conserved Ser179 residue in SDC4‐CD disrupted the formation of LLPS droplets, thereby inhibiting the association of syntenin with SDC4 on the PM. Moreover, upon treatment with a PKC inhibitor or bFGF, a protein that activates serine/threonine phosphatases, syntenin recruitment to the PM of HeLa cells was observed, suggesting that phosphorylation of SDC4 might abrogate the influence of protein LLPS in recruiting of syntenin on the PM. A phosphomimic mutant also showed that phosphorylation of Ser179 disrupted SDC4 LLPS droplet formation and inhibited the association between syntenin and SDC4 on the PM. Functionally, we found that condensate formation by SDC4 and syntenin was essential for syntenin sorting for packaging into exosomes, enhancing cell‐to‐cell communication, and that this process was abrogated by the phosphorylation of at SDC4 Ser179. Given that cells contain numerous transmembrane proteins that regulate multiple cellular pathways and in light of the profound functional effects of biomolecular condensation on exosome biogenesis, it will be of great interest to investigate the effect of protein phosphorylation and other posttranslational modifications on exosome biogenesis.

## 4 METHODS

### 4.1 Sample preparations

Peptides corresponding to the unphosphorylated (SDC4‐CD: RMKKKDEGSYDLGKKPIYKKAPTNEFYA) and phosphorylated cytoplasmic domain of SDC4 (p‐SDC4‐CD: RMKKKDEGpSYDLGKKPIYKKAPTNEFYA) which contain residues 171–198 of the human protein were synthesized using the standard Fmoc‐solid phase synthesis method. All synthesized peptides were identified by ESI‐MS (Thermo LTQ Orbitrap XL equipped with an electrospray ionization source), and their purities were assessed by an analytical HPLC.

### 4.2 Cell culture

HeLa cells were cultured in DMEM containing 10% FBS and 1% antibiotics (penicillin/streptomycin). The cells were cultured at 37°C with 5% CO_2_ in a humidified incubator.

### 4.3 Inhibitors and reagents

Phosphorylation of SDC4 was induced by PKC activator PMA (Beyotime) at the indicated concentrations. Dephosphorylation of SDC4 was induced by PKC inhibitor Staurosporine (Beyotime) at the indicated concentrations. bFGF (Sino Biological) activated Serine/tyrosine phosphatase and reduced SDC4 phosphorylation at the indicated concentrations. Cyanine 3 monosuccinimidyl ester [equivalent to Cy3® NHS ester] buys from AAT Bioquest.

### 4.4 Cy3 labelled SDC4‐CD in vitro

Add anhydrous DMSO into the vial of Cyanine 3 monosuccinimidyl ester to make a 10 mM stock solution. Mix well by pipetting or vortex. Kept from light and avoid freeze–thaw cycles. SDC4‐CD be dissolved in phosphate buffered saline (PBS), and adjust the pH to the range of 8.0–9.0 using 1 M sodium bicarbonate solution. For optimal labelling efficiency the final protein concentration range of 2–10 mg/mL. Add 5 μL of the dye stock solution (10 mM) into the vial of the protein solution (95 μL 10 mg/mL) with effective shaking. Continue to rotate or shake the reaction mixture at room temperature for 1 h. Then, it must be dialyzed against with PBS, to remove free Cyanine 3 monosuccinimidyl ester. In imaging assays, fluorescence labelled proteins were further diluted with the corresponding unlabelled proteins in the same buffer. Typically, for components in solution, the final ratio of fluorescence labelled protein: unlabelled protein was 3:100. The Cy3‐labelled SDC4‐CD sample was tested by LC–MS, and we found that only a part of SDC4‐CD were labelled with varying payloads of Cy3 (Supplementary Materials Figure S9).

### 4.5 Lipid bilayer preparation and phase transition assay

Phospholipids containing 98% POPC (Avantilipids), 2% DGS‐NTA‐Ni (Avantilipids) and were dried under a stream of nitrogen and resuspended by PBS to a final concentration of 0.5 mg/mL. The lipid solution was repeatedly frozen and thawed using a combination of liquid N_2_ and 37°C water bath until the solution turned clear. Then the solution a centrifugation at 14000 rpm for 1 h at 4°C. Supernatant containing SUVs was collected. 96 Well Glass Bottom Plate wash and lipid coating 96 Well Glass Bottom Plate (Cellvis) was initially washed with 5% Hellmanex II (Sigma) overnight, thoroughly rinsed with MilliQ H_2_O. The cover glass was then washed with 5 M NaOH for 1 h at 50°C and thoroughly rinsed with MilliQ H_2_O, repeated for three times, and followed by equilibration with phosphate‐buffered saline (PBS). Typically, 150 μL SUVs were added to a cleaned chamber and incubated for 2 h at 42°C, allowing SUVs to fully collapse on glass and fuse to form supported lipid bilayers (SLBs). SLBs were washed with PBS for three times to remove extra SUVs. Then it was blocked with the Cluster Buffer (the Protein Buffer supplied with 5 mg/mL BSA) for 1 h at room temperature. We used Cy3 labelled SDC4‐CD with an N‐terminal‐His6 tag (His‐SDC4‐CD), which attaching to DGS‐NTA‐Ni embedded in the lipid bilayers (Figure 1B). Initially, 100 μM His‐SDC4‐CD was added and incubated with SLBs for 3 h at 4°C temperature, followed by washing with PBS for three times to remove unbound His‐SDC4‐CD. 20 μM iFluor™ 488 labelled syntenin were premixed in PBS and then added to the His‐SDC4‐CD SLBs. All data were tested with LSCM.

### 4.6 Fluorescence recovery after photobleaching of cell membrane and in vitro condensates

Samples of SDC4 or SDC4 and syntenin phase separation in vitro or on lipid bilayers or on cell membrane were examined on Leica DMi8 microscope using a 100× objective (oil immersion). A single fluorescent droplet was bleached for 1.3 s with 50% laser power of a 488‐nm or 584 lasers (1 AU) respectively. After being photobleached, images were acquired at a rate of 2 s (on lipid bilayers) per frame for 35 or 2 s (on cell surface) for 200 s. Signals were normalized with pre‐bleached as 100% and 0 s after bleach as 0. At least three FRAP curves were averaged to produce each FRAP curve by Graphpad prism 7.0.

### 4.7 Analysis of the effect of salt concentration on SDC4 phase separation

Purified proteins (100 μM SDC4‐CD or different molar ratio) were desalted into increasing NaCl concentration assay buffer (50–450 mM) and mixed with (10% w/v) PEG‐8000. The mixed protein solution was immediately loaded into a 96‐well plate and incubated for 1 h at 4°C before imaging analysis. Images were captured with a Leica SP8 confocal microscopy with a ×100 objective (oil immersion) and LAS X software 3.2.

### 4.8 Analysis of the effect of 1,6‐Hexanediol on SDC4 phase separation

To test the effect of 1,6‐Hexanediol on SDC4 phase separation in vivo. 1,6‐hexanediol was diluted in cell culture media at 10% w/v. The culture media was replaced with media containing 1, 6‐hexanediol (10% w/v), and images were captured every 5 min with a Leica SP8 confocal microscope.

To test the effect of 1,6‐Hexanediol on SDC4 phase separation in vivo. Purified proteins (100 μM SDC4‐CD or different molar ratio) were desalted into phosphate‐buffer saline (PBS), then mixed with (10% w/v) PEG‐8000 and different concentration of 1, 6‐hexanediol (0%–10% w/v). Images were captured with a Leica SP8 confocal microscopy with a ×100 objective (oil immersion) and LAS X software 3.2.

### 4.9 Phase separation of SDC4/SDC4‐syntenin on cell membrane

The cells were plated on an eight‐well Lab‐Tek chambered coverglass at a density 3 × 10^4^ cells/well for HeLa cells in 200 μL medium and cultured for 24 h, the cells were transfected with pCDNA3.1‐SDC4 or pCDNA3.1‐SDC4‐IRES‐eGFP‐syntenin using Lipofectamine 8000 (Beyotime Biotechnology, China) for 24 h. For detection of cell membrane SDC4, the cells were incubated with an anti‐SDC4 antibody (Santa Cruz Biotechnology, USA, 1:200 dilution) for 2 h at 37°C with 5% CO_2_ in a humidified incubator. After three washing steps with PBS, the cells were incubated with the cells were incubated with Cy3‐labelled Goat Anti‐Mouse IgG(H+L) (Beyotime Biotechnology, China, 1:1000 dilution) antibody for 2 h at 37°C with 5% CO_2_ in a humidified incubator. If only detected eGFP‐syntenin, the cells did not incubate with antibody. The cells were examined under a confocal laser scanning microscopy using an inverted Leica SPi8 microscope, equipped with lasers for 488‐nm, 584 nm excitation. Images were acquired using a 100× objective.

### 4.10 Immunofluorescence

The cells were plated on an eight‐well Lab‐Tek chambered coverglass at a density 3 × 10^4^ cells/well for HeLa cells in 200 μL medium and cultured for 24 h, the cells were transfected with pCDNA3.1‐SDC4 or pCDNA3.1‐SDC4‐IRES‐eGFP‐syntenin using Lipofectamine 8000 (Beyotime Biotechnology, China) for 24 h. Then cells were fixed for 15 min with 4% paraformaldehyde, washed, and incubated in blocking buffer for 1 h (1× PBS with 3% BSA). After washed with PBS for three times, cells were incubated in 1× PBS with 0.5% Triton X‐100 for 10 min. For detection of cell membrane SDC4, the cells were incubated with an anti‐SDC4 antibody (Santa Cruz Biotechnology, USA) diluted 1:200 in medium containing 3% BSA for 2 h at room temperature. After three washing steps with PBS, the cells were incubated with the cells were incubated with Cy3‐labelled Goat Anti‐Mouse IgG(H+L) (Beyotime Biotechnology, China, 1:1000 dilution) antibody for 1 h at room temperature in the dark. The cells were examined under a confocal laser scanning microscopy using an inverted Leica SPi8 microscope, equipped with lasers for 488‐nm, 584 nm excitation. Images were acquired using a 100× objective.

### 4.11 PMA/staurosporine/bFGF regulate SDC4 phase separation and the recruitment of syntenin on the plasma membrane

The cells were plated on an six‐well cell culture plate at a density 40 × 10^5^ cells/well for HeLa cells in 2 mL medium or an eight‐well Lab‐Tek chambered coverglass at a density 3 × 10^4^ cells/well for HeLa cells in 200 μL medium and cultured for 24 h, the cells were transfected with pCDNA3.1‐SDC4 or pCDNA3.1‐SDC4‐IRES‐eGFP‐syntenin using Lipofectamine 8000 (Beyotime Biotechnology, China) for 24 h. Then, the transfected cells were incubated with different concentrations of PMA (0.1, 1, 10 μM) or staurosporine (0.01, 0.1, 1 μM) for 30 min. The subcellular localization of SDC4 or syntenin were examined under a confocal laser scanning microscopy and the level of SDC4 phosphorylation were examined by anti P‐SDC4 antibody (Catalogue Number: AF8061, Affinity Biosciences) with western blot.

### 4.12 Syntenin exosome extraction and identification

The transfected SDC4‐syntenin were seeded in the no FBS content DMEM culture medium, and cultured in an incubator with 5% CO_2_ at 37°C. After 72 h, the cell supernatant was collected and centrifugation was carried out to remove cell debris. The exosomes were extracted on the basis of the instructions of Hieff Quick exosome isolation kit (41201ES50, Yeasen Company, Shanghai, China). The cell supernatant and separation reagent of exosome were added into the centrifuge tube at a ratio of 4: 1 and vortex oscillated for 1 min, incubated at 4°C for 2 h. Next, the samples were centrifuged at 18000*g* at 4°C for 2 h, followed by the removal of the supernatant, the precipitates were collected. The samples were resuspended in 80 μL PBS in an EP tube and centrifuged at 12000*g* at 4°C for 2 min. Collected the supernatant and stored at −80°C.

### 4.13 Transmission electron microscopy (TEM)

Took 10 μL of exosome suspension dropped onto the copper grid with carbon film for 5 min, and then use filter paper to absorb the excess liquid (the unstained sample can observed under TEM and take images). Then, dropped 2% phosphotungstic acid on the copper grid to stain for 2 min, use filter paper to absorb excess liquid, and dry at room temperature. The cuprum grids are observed under TEM and take images.

### 4.14 Western blot analysis

The exosome samples (50 μL) added 10 μL RIPA lysis buffer, mixed, and placed on ice for 30 min. Subsequently, mixed with 20 μL of 4× SDS loading buffer with DTT (reducing) respectively, heated for 5 min at 100°C and analysed with western blot.

Anti‐eGFP antibody (Beyotime Biotechnology, China, 1:1000 dilution) as used for the detection of syntenin in exosome or cell lysates. Anti‐CD63 antibody (abcam, Britain, 1:1000 dilution) as used for the detection of CD63 in exosome. Anti‐SDC4 antibody (Santa Cruz Biotechnology, USA, 1:200 dilution) as used for the detection of SDC4 in cell lysates. Anti‐vinculin antibody (Santa Cruz Biotechnology, USA, 1:200 dilution) as used for the detection of vinculin in cell lysates. Horseradish peroxidase‐linked anti mouse IgG (Beyotime Biotechnology, China) was used as secondary antibody (1:2000 dilution). The signals were developed using BeyoECL Plus regent (Beyotime Biotechnology, China) and imaged with Chemiscope miniimaging system (CLINX, China). The results were analysed with ImageJ 1.53.

### 4.15 Wound‐healing assay

The cells were seeded in 6 well plate at a density 3 × 10^5^ cells/well for HeLa cells in 2 mL medium and cultured for 24 h, the cells were transfected with pCDNA3.1‐SDC4‐IRES‐eGFP‐syntenin/pCDNA3.1‐SDC4^S‐E^‐IRES‐eGFP‐syntenin/pCDNA3.1‐SDC4^S‐A^‐IRES‐eGFP‐syntenin, respectively, using Lipofectamine 8000 (Beyotime Biotechnology, China) for 24 h. Images were taken at 0, 24, 48 and 72 h with optical microscopy. The wound‐healing distance was detected by Photoshop and the results were analysed with Graphpad prism 7.0.

### 4.16 CRISPR/Cas9 mediated eGFP KI cell lines

CRISPR/Cas9 was used to generate endogenously‐mEGFP‐tagged SDC4 in HeLa cells. Oligos coding for guide RNAs targeting the N terminus of SDC4 was 5′‐TCGCCGAGTCGGTGGGTGCTG‐3′. The donor plasmid contained eGFP flanked by ~1000 bases upstream of eGFP insertion site and ~1000 bases downstream (VectorBuilder, China). Donor and guide plasmids were transfected into cells at a 1:1 molar ratio using Lipofectamine 8000 (Beyotime Biotechnology, China). Cells were grown in puromycin‐containing (0.5 μg/mL) medium for 3 days, and eGFP positive single cell clones were selected by single‐cell cloned, then verified by sequencing and colonies were picked 14 days after seeding into 96 well plates. Cells were further imaging by Confocal laser scanning microscopy.

## AUTHOR CONTRIBUTIONS

*Supervision*: S.Z.L, Y.X.C. *Methodology*: T.Z. *Data analysis*: S.Z.L, Y.X.C, T.Z, X.L.Y, G.F.D, K.F.H. *Funding acquisition*: S.Z.L. *Writing*: S.Z.L, Y.X.C, T.Z.

## FUNDING INFORMATION

This work was supported by funding from National Key R&D Program of China (2021YFC2103900), National Natural Science Foundation of China (22261132513, 22277009, 92353302, 22177059), Tsinghua University Initiative Scientific Research Program, Joint Project of BRC‐BC (Biomedical Translational Engineering Research Center of BUCT‐CJFH) (XK2023‐14, XK2022‐07).

## CONFLICT OF INTEREST STATEMENT

The authors declare that they have no conflict of interest.

## Supporting information


**Data S1.** Supporting Information.

## Data Availability

The data that support the findings of this study are available from the corresponding author upon reasonable request.
