# Supplementary material for: Phosphorylation‐regulated phase separation of syndecan‐4 and syntenin promotes the biogenesis of exosomes
Source: Cell Prolif. 2024 Apr 11;57(10):e13645. doi: 10.1111/cpr.13645 (PMC11471451; doi:10.1111/cpr.13645)
Supplement: Supplementary file 1 — Data S1. Supporting Information. [file CPR-57-e13645-s001.docx]

**Supplemental information**

**Phosphorylation-regulated phase separation** **of syndecan-4 and syntenin promotes the biogenesis of exosomes**

**Authors**

Tian Zhao^1^, Xiaolan Yang^1^, Guangfei Duan^1^, Jialin Chen^1^, Kefeng He^1^, Yong-Xiang Chen^2*^, Shi-Zhong Luo^1*^

**Affiliations**

^1^State Key Laboratory of Chemical Resource Engineering, College of Life Science and Technology, Beijing University of Chemical Technology, Beijing, 100029, China.

^2^Key Laboratory of Bioorganic Phosphorus Chemistry and Chemical Biology (Ministry of Education), Department of Chemistry, Tsinghua University, Beijing 100084, China

***Corresponding authors**

Correspondence to Dr. Shi-Zhong Luo: [luosz@mail.buct.edu.cn](mailto:luosz@mail.buct.edu.cn)

Dr.Yong-Xiang Chen: chen-yx@mail.tsinghua.edu.cn

**Supplementary Materials Fig. S1
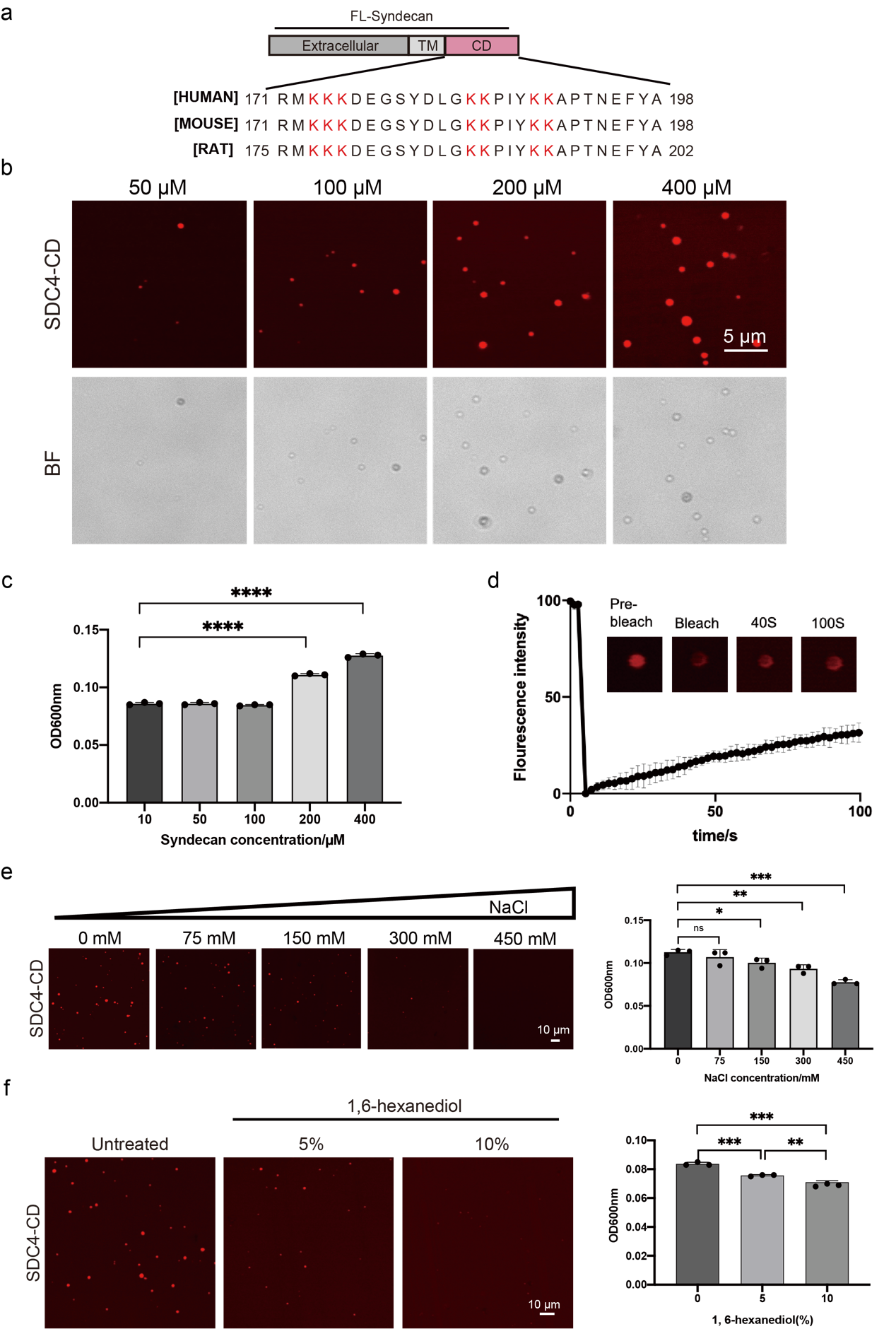
**

**Supplementary Materials Fig. S1**

**SDC4-CD formed phase separation *in vitro***

**a** Alignment of human, mouse, and rat SDC4-CD domains. **b** Confocal microscopy images showing the assembly of 50 μM-400 μM Cy3-labelled SDC4-CD with 10% PEG. BF, bright field. Scale bar = 5 µm. **c** Turbidity measurement of Cy3-labelled SDC4-CD with 10% PEG; n = 3 biologically independent samples, and the data are presented as the mean values ± SEMs. Comparisons among groups were performed via ordinary one-way ANOVA. **** P<0.0001. **d** ﻿FRAP assay results showing the droplets formed by Cy3-labelled SDC4-CD with 10% PEG; n = 3 biologically independent samples, and the data are presented as the mean values ± SEMs. **e** Confocal microscopy images showing that high NaCl concentrations weakened Cy3-labelled SDC4-CD phase separation. Cy3-labelled SDC4-CD (100 μM) was mixed with 10% PEG and NaCl at the indicated concentrations. Turbidity measurement of Cy3-labelled SDC4-CD with 10% PEG and NaCl at the indicated concentrations; n = 3 biologically independent samples, and the data are presented as the mean values ± SEMs. Comparisons among groups were performed via ordinary one-way ANOVA. * P=0.0286, ** P=0.004, *** P=0.001. **f** Confocal microscopy images showing that 1,6-hexanediol weakened Cy3-labelled SDC4-CD phase separation. Cy3-labelled SDC4-CD (100 μM) was mixed with 10% PEG and the indicated 1,6-hexanediol concentrations. Turbidity measurement of Cy3-labelled SDC4-CD with 10% PEG and 1,6-hexanediol at the indicated concentrations; n = 3 biologically independent samples, and the data are presented as the mean values ± SEMs. Comparisons among groups were performed via ordinary one-way ANOVA. ** P=0.0022, *** P=0.0004 (5% 1,6-hexanediol vs. 0% 1,6-hexanediol), *** P=0.0001 (10% 1,6-hexanediol vs. 0% 1,6-hexanediol).**Supplementary Materials Fig. S2**

**
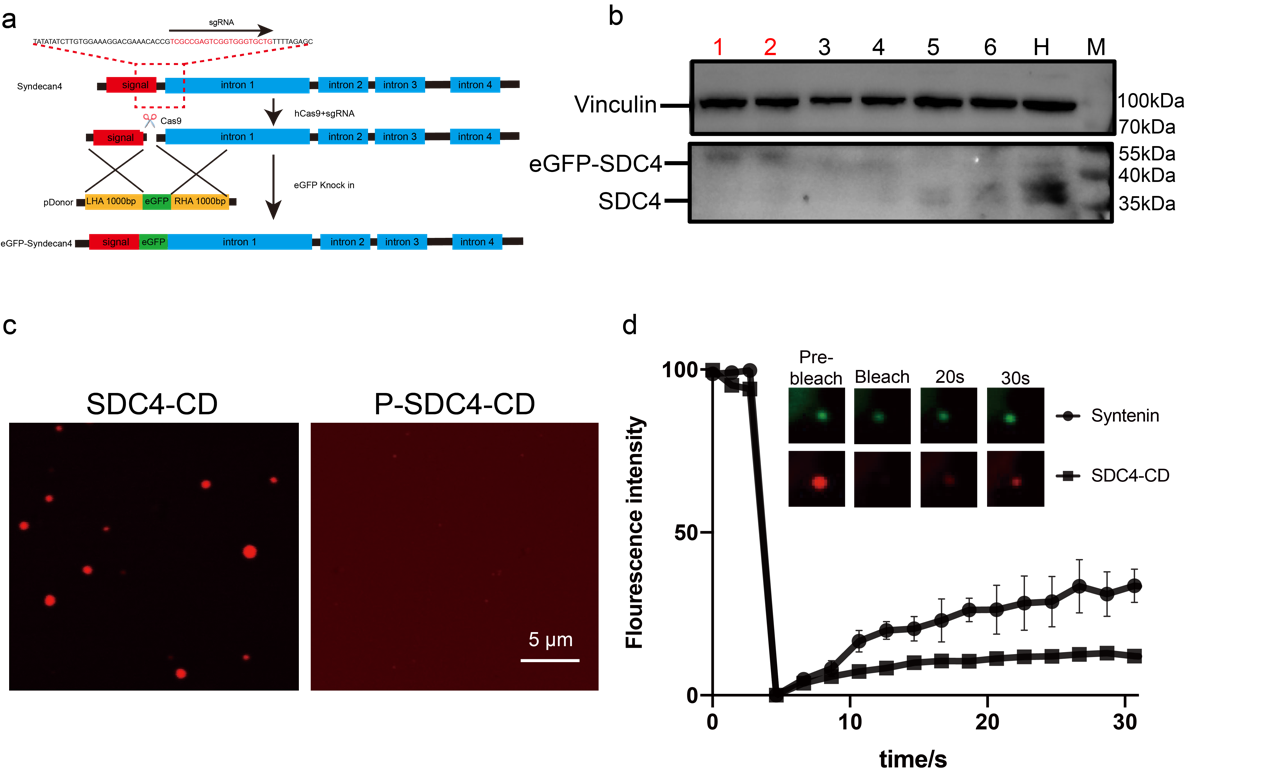
**

**Supplementary Materials Fig. S2**

**Phosphorylated weakened SDC4 phase separation and** **interfered with the recruitment of Syntenin to the PM**

**a** Design of sgRNAs for eGFP-Tag knock-in; **b** Western blots of SDC4 for single cell clones from (**a**). **c** Confocal microscopy images showing that phosphorylated weakened Cy3-labelled SDC4-CD phase separation. Cy3-labelled SDC4-CD (100 μM) and Cy3-labelled P-SDC4-CD (100 μM) was mixed with 10% PEG respectively**. d** FRAP assay results showing droplets formed by SDC4-CD-Syntenin on structured lipid bilayers (SLBs). The curve represents the average of the signals from 3 droplets.

**Supplementary Materials Fig. S3**

**
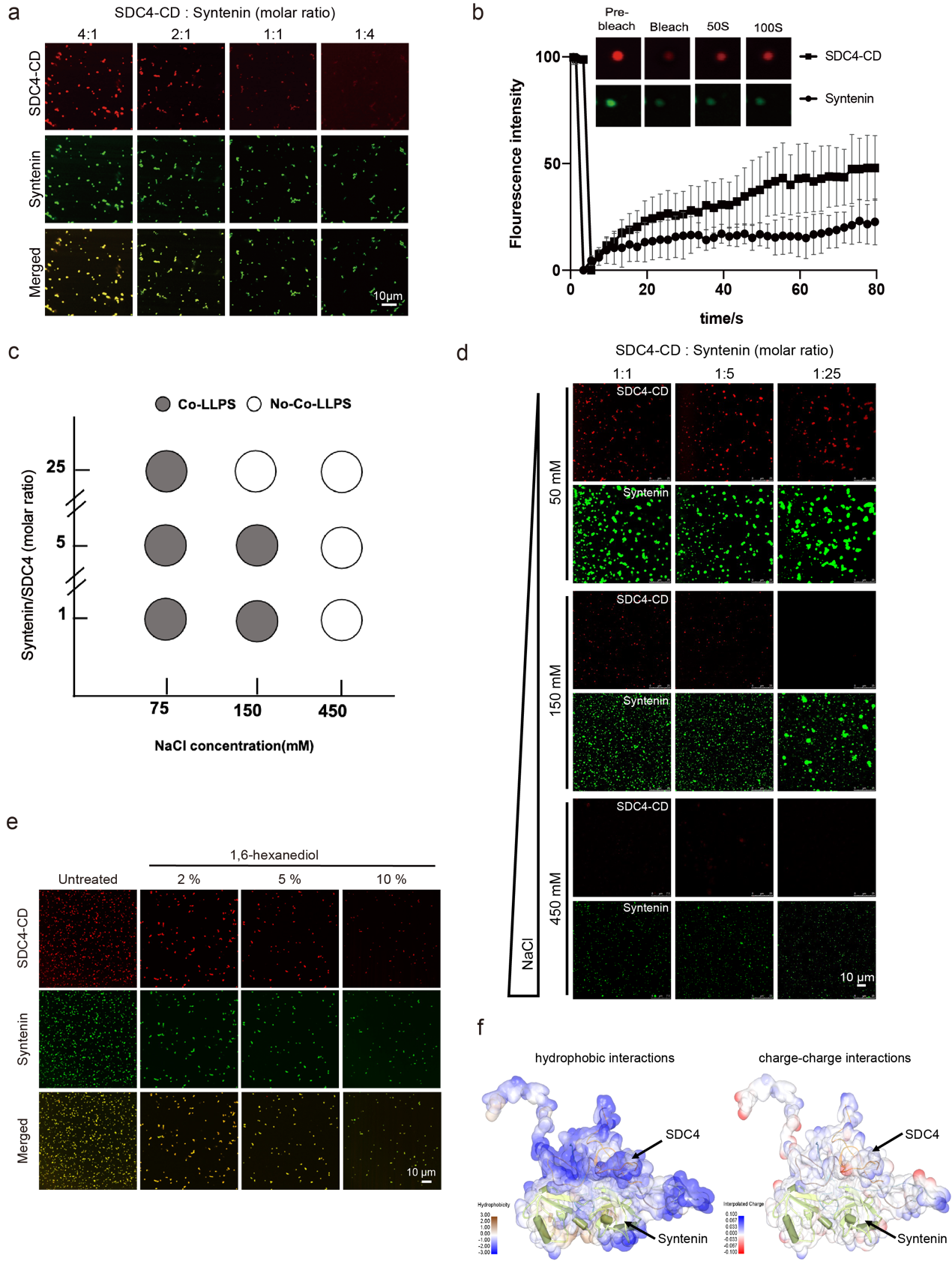
**

**Supplementary Materials Fig. S3**

**SDC4-CD phase separation recruited Syntenin to droplets.**

**a** ﻿Confocal microscopy images showing the assembly of 20 µM iFluor^TM^ 488-labelled Syntenin mixed with Cy3-labelled SDC4-CD at different ratios. Scale bar = 10 µm. **b** FRAP assay results showing the droplets formed by Cy3-labelled SDC4-CD: iFluor^TM^ 488-labelled Syntenin = 4:1; n = 3 biologically independent samples, and the data are presented as the mean values ± SEMs. **c** Statistical analysis showing that high NaCl concentrations weakened SDC4-CD and Syntenin phase separation. **d** Confocal microscopy images showing that high NaCl concentrations weakened SDC4-CD and Syntenin phase separation. iFluor^TM^ 488-labelled Syntenin (20 µM) was mixed with 80 µM Cy3-labelled SDC4-CD at the indicated NaCl concentrations. **e** Confocal microscopy images showing that 1,6-hexanediol weakened SDC4-CD and Syntenin phase separation *in vitro*. Then, 80 μM Cy3-labelled SDC4-CD and 20 µM iFluor^TM^ 488-labelled Syntenin were mixed with 10% PEG and 1,6-hexanediol at the indicated concentrations. **f** A GROMACS molecular dynamics simulation predicted that SDC4-CD recruits Syntenin on the basis of charge‒charge and hydrophobic interactions.

**Supplementary Materials Fig. S4**

**
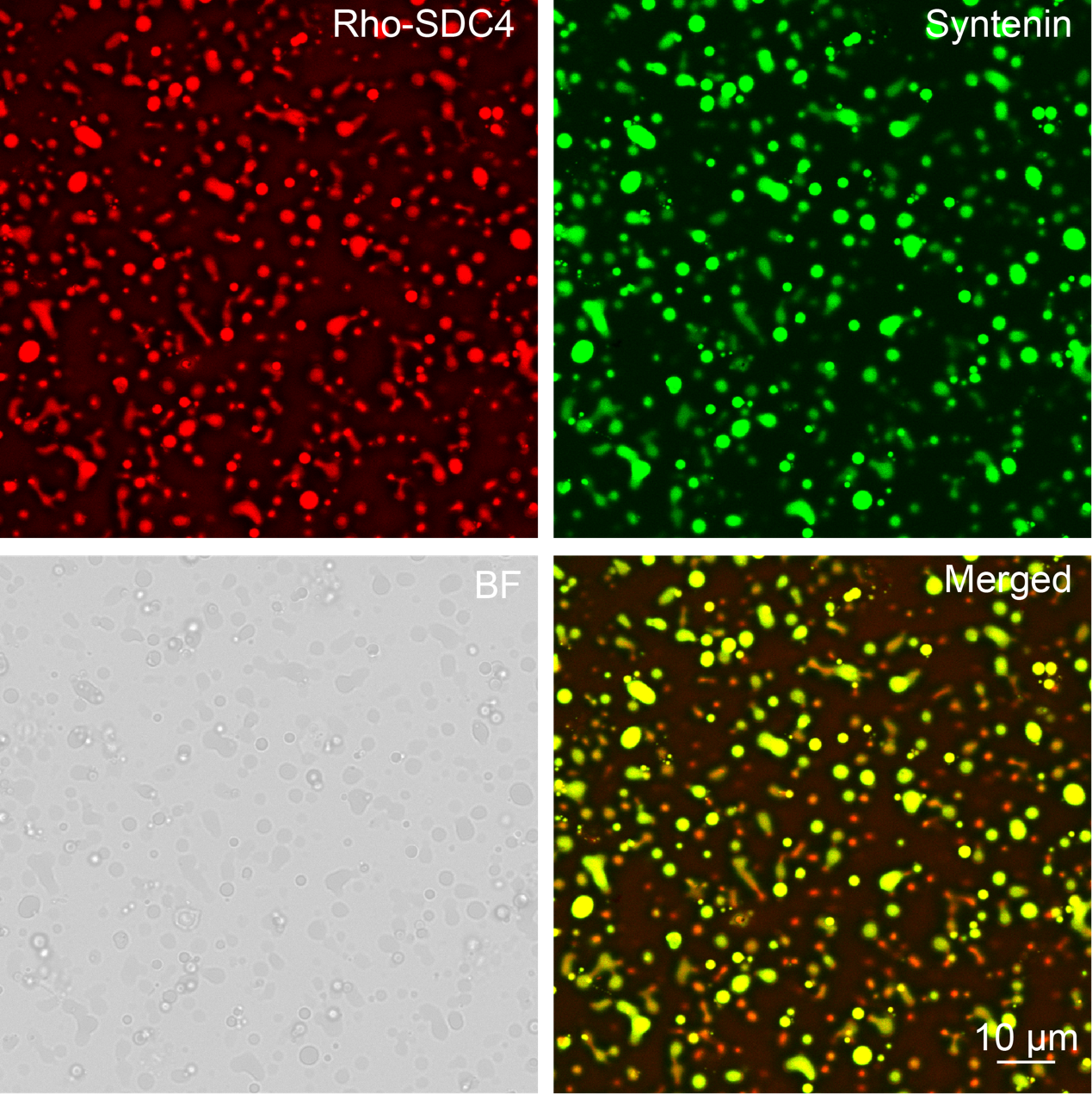
**

**Supplementary Materials Fig. S4**

**Rho-labelled SDC4-CD phase separation with syntenin.** Confocal laser scanning microscopy (CLSM) images showing the assembly of iFluor^TM^ 488-labelled syntenin mixed with Rho-labelled SDC4-CD.

**Supplementary Materials Fig. S5**

**
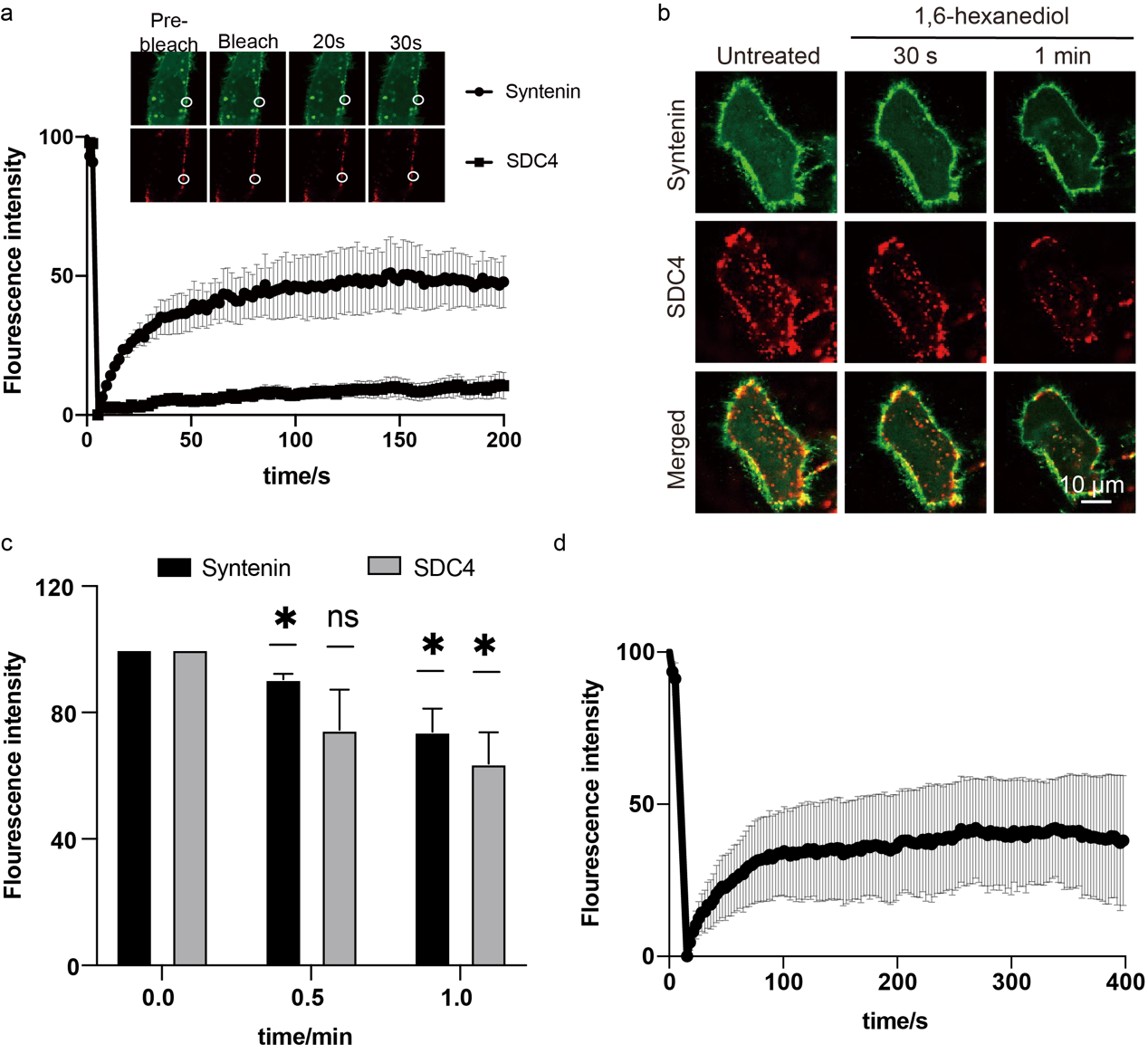
**

**Supplementary Materials Fig. S5**

**FRAP assays** **proved that SDC4-CD with** **Syntenin underwent phase separation and that the resulting droplets were homogeneous and fluid.**

**a** FRAP assay results showing the fluorescence recovery of SDC4-CD-Syntenin cophase separation on the cell membrane; n = 3 biologically independent samples, and the data are presented as the mean values ± SEMs. **b** Confocal microscopy images showing that 1,6-hexanediol weakened SDC4-CD and Syntenin phase separation on the PM. **c** Quantification of the fluorescence intensity of the condensate in the cell after treatment with 10% 1,6-hexanediol in a time-dependent manner. * P<0.05. **d** Without an antibody recognizing SDC4, the FRAP assay showing the fluorescence recovery of eGFP-Syntenin on the plasma membrane.

**Supplementary Materials Fig. S6**

**
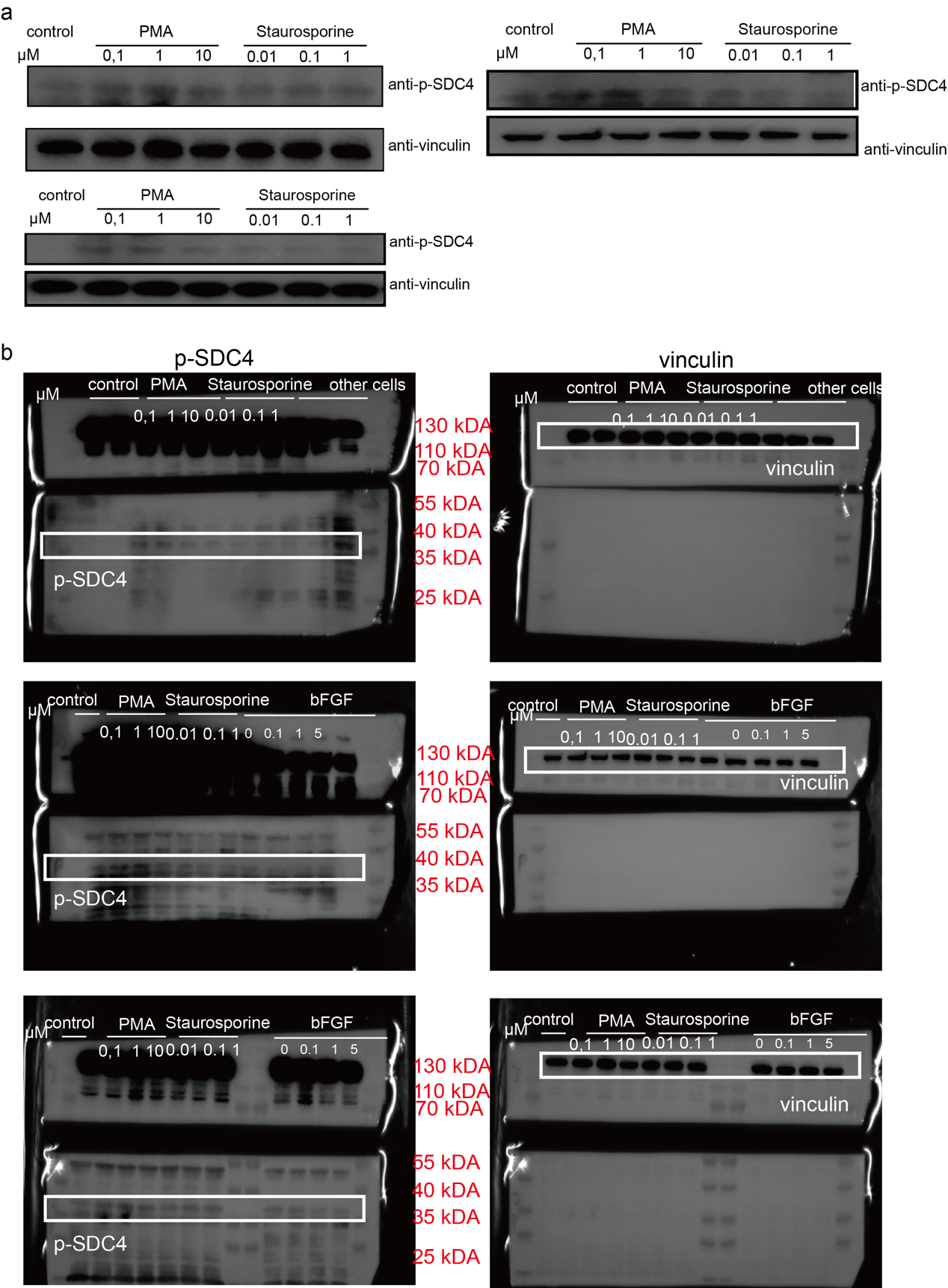
**

**Supplementary Materials Fig. S6**

**The three replicated experiments and the** **raw data of Fig. 3**

**a,** Three replicated results of western blot. Western blot showed that PMA and staurosporine regulated the phosphorylation level of SDC4. **b,** Three replicated raw data of western blot.

**Supplementary Materials Fig. S7**


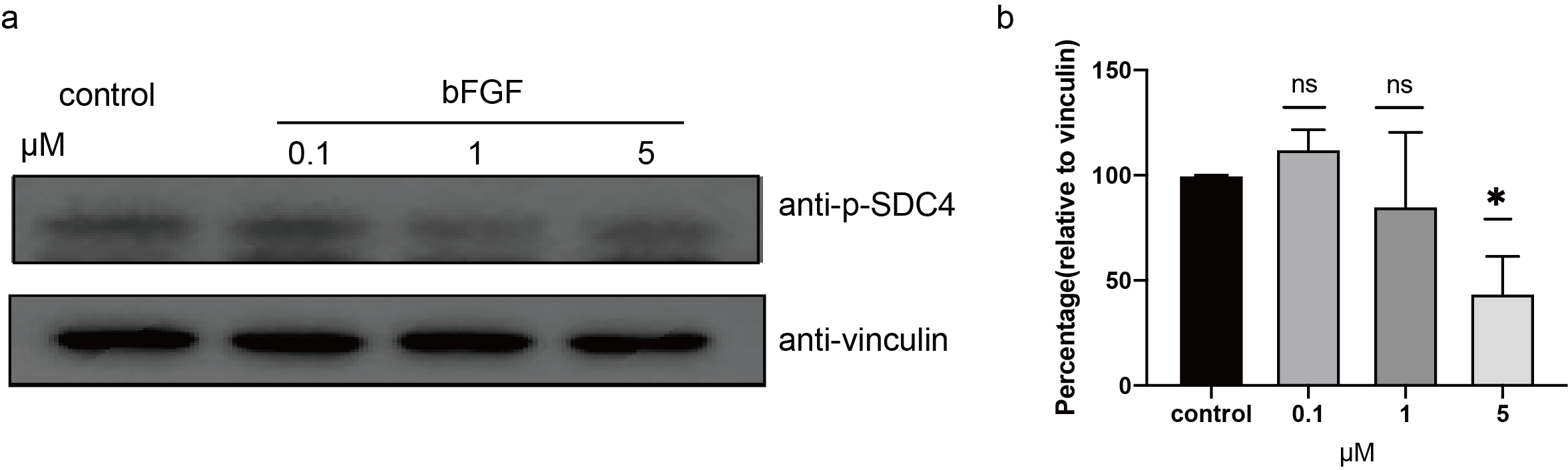


**Supplementary Materials Fig. S7**

**Western blot showed that bFGF regulated the phosphorylation level of SDC-4.**

**a** Western blot showing that bFGF decreased the phosphorylation level of SDC-4 with the increased concentration. **b** Quantification of the level of SDC4 phosphorylation. *P=0.0462.

**Supplementary Materials Fig. S8**


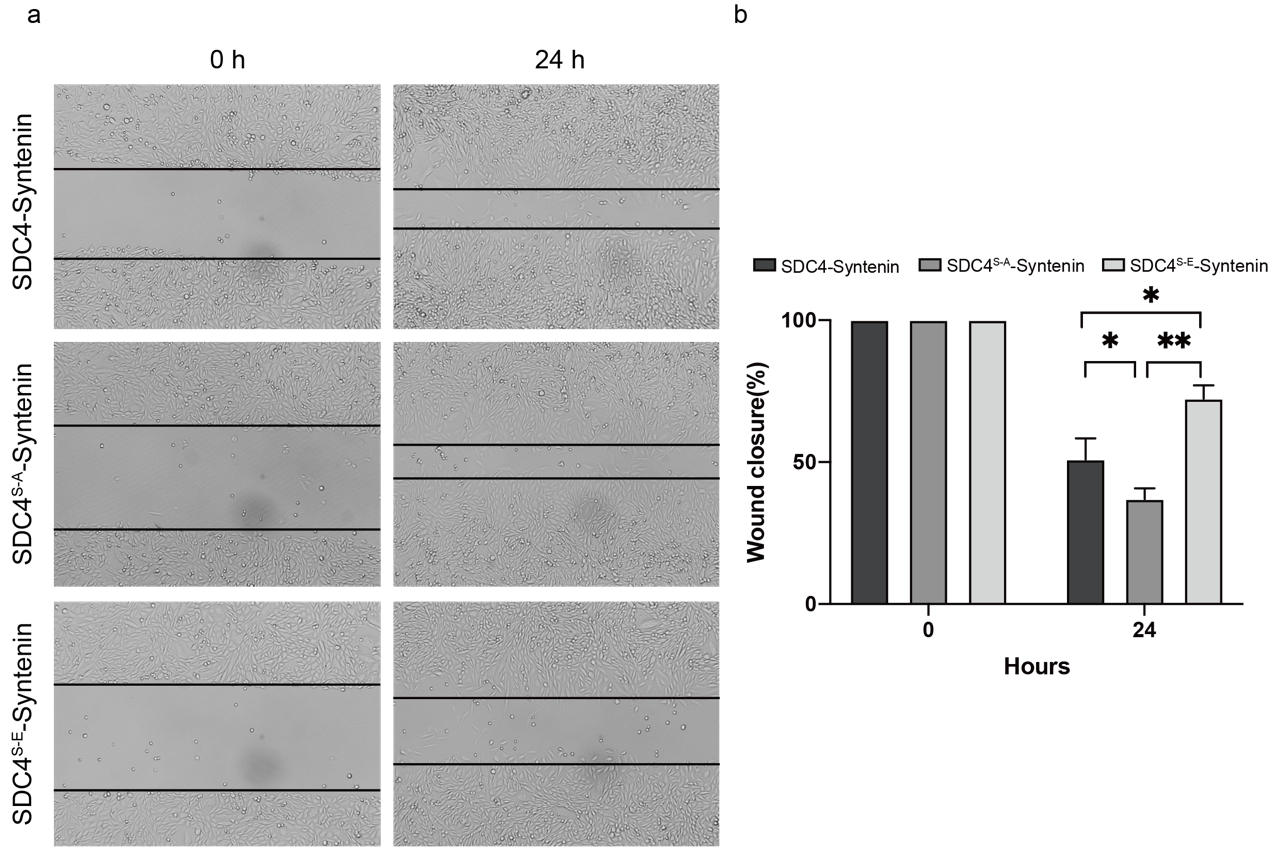


**Supplementary Materials Fig. S8**

**The dephosphorylation-mimicking mutant SDC4^S-A^-syntenin improved CHO cells viability.**

**a** Percentage of the wound closed after SDC4-syntenin, SDC4^S-A^-syntenin or SDC4^S-E^-Sy overexpression. ﻿Micrographs were taken immediately after wounding and 24 h after the introduction of a wound. Black lines denote wound edges. **b** ﻿Values significantly different from controls are indicated with an asterisk (*P<0.05, **P<0.01).

**Supplementary Materials Fig. S9**


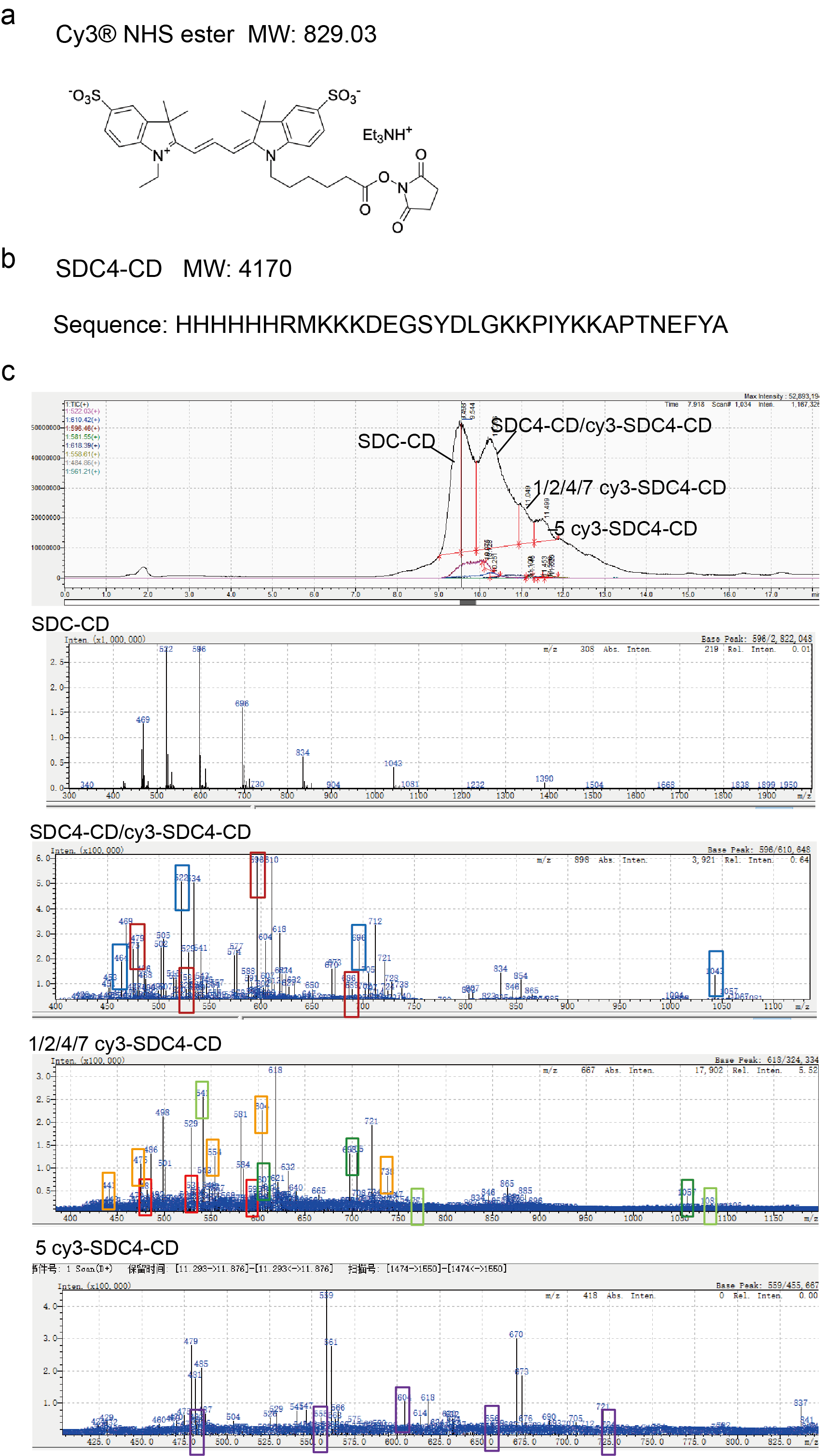


**Supplementary Materials Fig. S9 in revised manuscript**

**The LC-MS results of** **Cy3-labeled SDC4-CD sample.**

**a** Cy3® NHS ester structure and molecular weight. **b** SDC4-CD sequence and molecular weight. **c** TIC and m/z of Cy3 labeled SDC4-CD. Different colored frames represent SDC4-CD connected to different numbers of Cy3. Red represents one Cy3 to label SDC4-CD; light green represents two Cy3 to label SDC4-CD; blue represents four Cy3 to label SDC4-CD; purple represents five Cy3 to label SDC4-CD; dark green represents five Cy3 to label SDC4-CD.
